# 0992. Effects of inhaled aerosolized insulin on acutely injured lungs under normoglycemia: insulin may contribute to enhance alveolar liquid clearance through epithelial sodium channel expression

**DOI:** 10.1186/2197-425X-2-S1-P77

**Published:** 2014-09-26

**Authors:** M Senda, W Fan, K Nakazawa, K Makita

**Affiliations:** Dept. of Anesthesiology & Critical Care Medicine, Tokyo Medical and Dental University, Tokyo, Japan; Dept. of Anesthesia, Inner Mongolia People's Hospital, Inner Mongolia, China

## Introduction

We have previously shown that hyperglycemia enhances inflammatory responses and that aerosolized insulin ameliorates inflammatory responses compared to identical doses of intravenous insulin in an experimental model of acute lung injury [1].

## Objectives

The purpose of the present study is to clarify whether insulin exerts anti-inflammatory effects in acute lung injury under normoglycemia.

## Methods

Twenty mechanically ventilated rabbits were randomly allocated into control group (group-C: n=10) or inhaled aerosolized insulin group (group-I: n=10). They were anesthetized with intramuscular ketamine and pentobarbital. Mechanical ventilation was initiated after tracheostomy (TV=10ml/kg, RR=25/min, I:E ratio=1:2, PEEP=3cmH_2_O and F_I_O_2_=1.0). Lung lavage was performed with warm normal saline (60 ml) to produce lung injury. After induction of acute lung injury, the ventilator settings were changed as follows: TV=6 ml/kg, RR=30-40/min, and PEEP=10cmH_2_O. The group-I received aerosolized insulin (1 IU/kg) diluted in 5ml normal saline through an ultrasonic nebulizer every three hours while the group-C received 5ml of aerosolized normal saline. Arterial blood samples were obtained for blood glucose and blood gas analyses at 60, 120, 180, 240, and 360 min after ventilation settings were changed. After 6 hours of treatment, the lungs and heart were excised en bloc, and then we examined interleukin-8 (IL-8), toll-like receptor 4 (TLR-4) mRNA expressions in bronchoalveolar lavage fluid (BALF) cells. We also examined mRNA expressions of endothelial sodium channel alpha subunit (ENaCα), serum/glucocorticoid regulated kinase 1 (SGK1), transforming growth factor-β1 (TGF-β1) and keratinocyte growth factor (KGF) in the lung tissue so as to examine whether insulin contributes to recovery from lung injury.

## Results

**Figure 1:**
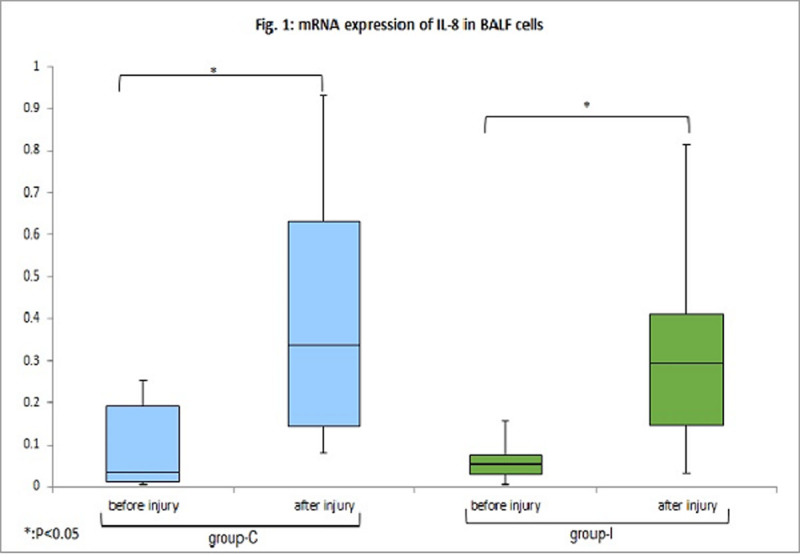
mRNA expressions of IL-8 in BALF cells.

**Figure 2:**
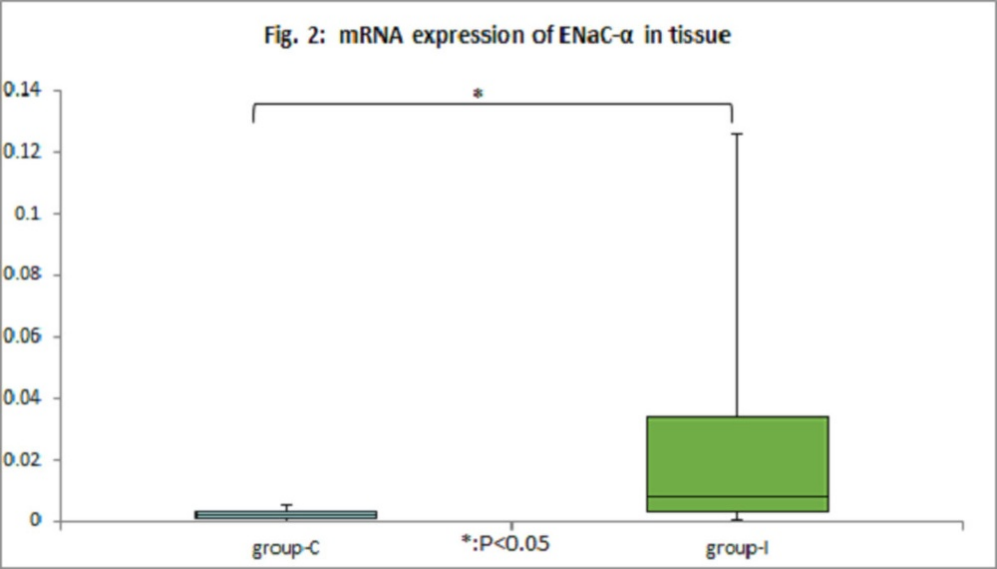
mRNA expression of ENaC-α in tissue.

The blood glucose levels maintained within normal limits (80-180mg/dl) and identical between the two groups throughout the experimental course. The mRNA expressions of IL-8 in the BALF cells elevated after injury, however the levels were not different between group-C and group-I (Fig. 1). The mRNA expression of TLR-4 in BALF cells did not elevate after experiment. The tissue levels of mRNA expression of ENaCα were significantly higher in the group-I (Fig.2). However, we could not find any difference in mRNA expression of TGF-β1 and KGF in the tissue of both groups.

## Conclusions

The results failed to show insulin ameliorate inflammatory responses in acute lung injury under normoglycemia. This may partly explained by the low tidal volume ventilation with PEEP protected exacerbation of inflammatory responses in this injury model. However we found that insulin might contribute to the lung fluid clearance via expression of epithelial sodium channel.
